# Changes in the midpalatal and pterygopalatine sutures induced by micro-implant-supported skeletal expander, analyzed with a novel 3D method based on CBCT imaging

**DOI:** 10.1186/s40510-017-0188-7

**Published:** 2017-11-01

**Authors:** Daniele Cantarella, Ramon Dominguez-Mompell, Sanjay M. Mallya, Christoph Moschik, Hsin Chuan Pan, Joseph Miller, Won Moon

**Affiliations:** 1 Division of Oral Biology and Medicine, School of Dentistry, Center for Health Science, University of California, 10833 Le Conte Avenue, Box 951668, Los Angeles, CA 90095-1668 USA; 2 Division of Growth and Development, Section of Orthodontics, School of Dentistry, Center for Health Science, University of California, Room 63-082 CHS, 10833 Le Conte Avenue, Box 951668, Los Angeles, CA 90095-1668 USA; 3 Division of Diagnostic and Surgical Sciences, Section of Oral and Maxillofacial Radiology, School of Dentistry, Center for Health Science, University of California, Room 53-068 B CHS, 10833 Le Conte Avenue, Box 951668, Los Angeles, CA 90095-1668 USA; 4 Division of Integrative Anatomy, Department of Pathology and Laboratory Medicine, Geffen School of Medicine, Center for Health Science, University of California, Room 52-068 CHS, 10833 Le Conte Avenue, Box 951668, Los Angeles, CA 90095-1668 USA

**Keywords:** Cone beam computed tomography (CBCT), Midpalatal suture, Palatal expansion, Pterygopalatine suture

## Abstract

**Background:**

Mini-implant-assisted rapid palatal expansion (MARPE) appliances have been developed with the aim to enhance the orthopedic effect induced by rapid maxillary expansion (RME). Maxillary Skeletal Expander (MSE) is a particular type of MARPE appliance characterized by the presence of four mini-implants positioned in the posterior part of the palate with bi-cortical engagement. The aim of the present study is to evaluate the MSE effects on the midpalatal and pterygopalatine sutures in late adolescents, using high-resolution CBCT. Specific aims are to define the magnitude and sagittal parallelism of midpalatal suture opening, to measure the extent of transverse asymmetry of split, and to illustrate the possibility of splitting the pterygopalatine suture.

**Methods:**

Fifteen subjects (mean age of 17.2 years; range, 13.9–26.2 years) were treated with MSE. Pre- and post-treatment CBCT exams were taken and superimposed. A novel methodology based on three new reference planes was utilized to analyze the sutural changes. Parameters were compared from pre- to post-treatment and between genders non-parametrically using the Wilcoxon sign rank test. For the frequency of openings in the lower part of the pterygopalatine suture, the Fisher’s exact test was used.

**Results:**

Regarding the magnitude of midpalatal suture opening, the split at anterior nasal spine (ANS) and at posterior nasal spine (PNS) was 4.8 and 4.3 mm, respectively. The amount of split at PNS was 90% of that at ANS, showing that the opening of the midpalatal suture was almost perfectly parallel antero-posteriorly. On average, one half of the anterior nasal spine (ANS) moved more than the contralateral one by 1.1 mm. Openings between the lateral and medial plates of the pterygoid process were detectable in 53% of the sutures (*P* < 0.05). No significant differences were found in the magnitude and frequency of suture opening between males and females. Correlation between age and suture opening was negligible (*R* ^2^ range, 0.3–4.2%).

**Conclusions:**

Midpalatal suture was successfully split by MSE in late adolescents, and the opening was almost perfectly parallel in a sagittal direction. Regarding the extent of transverse asymmetry of the split, on average one half of ANS moved more than the contralateral one by 1.1 mm. Pterygopalatine suture was split in its lower region by MSE, as the pyramidal process was pulled out from the pterygoid process. Patient gender and age had a negligible influence on suture opening for the age group considered in the study.

## Background

The undesirable side effects in conventional rapid maxillary expansion (RME) are limited skeletal movement, dentoalveolar tipping, root resorption, detrimental periodontal consequences, and lack of long-term stability [1]. To moderate these effects, clinicians in recent years have utilized micro-implant-assisted rapid palatal expansion (MARPE) [2]. The Maxillary Skeletal Expander (MSE) is a particular type of MARPE appliance that differs from the others because it promotes bi-cortical engagement of the four micro-implants into the cortical bone of the palate and nasal floor [3–5].

Due to the higher interdigitation of the midpalatal suture after puberty, some authors affirm that expansion of the maxilla in post-pubertal patients is not feasible [6] and surgically assisted rapid palatal expansion (SARPE) is needed [7, 8]. However, recent evidence has suggested that a successful expansion of the midpalatal suture in late adolescents can be possible with bone-borne and tooth-borne palatal expanders [3, 4, 9]. Although MARPE appliances have been developed with the aim of enhancing the orthopedic effect of maxillary expansion, comparisons between tooth-borne and bone-borne expanders led to different results. In fact, Lin et al. [9] found greater skeletal expansion with a bone-borne appliance, while Lagravère et al. [2] reported that the two types of expanders generate similar skeletal effects.

In regard to the pterygopalatine suture, it has been shown that the attempt to disarticulate it in dry skulls in late juvenile and early adolescent periods is always accompanied by fracture of heavily interdigitated osseous surfaces, as a result of rigid interdigitation and high resistance to separation of articulating bones [10]. Ghoneima et al. in a clinical study conducted with CBCT imaging in early adolescents concluded that this suture cannot be split when tooth-borne palatal expanders are utilized [11]. To the authors’ knowledge, no investigation has been conducted on the modifications induced on the pterygopalatine suture by bone-borne maxillary expanders.

Traditionally, analysis of the midpalatal suture during rapid palatal expansion was conducted using study models [1, 12], two-dimensional imaging [12–14], and, more recently, three-dimensional imaging based on computed tomographic data [15–18]. The introduction of cone beam computed tomography (CBCT) in the orthodontic field and the development of new computer software allows for multiplanar, 3-dimensional (3D) reconstructions, and, thus, more possibilities in diagnosis of the craniofacial complex in living subjects [19, 20].

The aim of the present study is to investigate the effects on midpalatal and pterygopalatine sutures induced by a bone-borne expander (MSE) in late adolescents, using high-resolution CBCT. Specific aims are to define the magnitude and sagittal parallelism of midpalatal suture opening, to measure the extent of transverse asymmetry of split, and to illustrate the possibility of splitting the pterygopalatine suture.

## Methods

### Study design

The present retrospective study received approval from the Institutional Review Board (IRB) at University of California, Los Angeles (UCLA).

### Participants and intervention

The study included 15 consecutively debonded patients (6 males, 9 females) with a mean age of 17.2 ± 4.2 years (range 13.9–26.2 years) of dominant Hispanic ethnicity, who were treated with MSE (BioMaterials Korea, Inc.) and who met the inclusion criteria. Nine patients presented a bilateral posterior crossbite, five patients presented a unilateral crossbite, and one patient presented a maxillary transverse deficiency without dental crossbite. All patients were treated at the Orthodontic Clinic of the UCLA School of Dentistry. Treatment with MSE was started and completed before bonding of orthodontic brackets in all patients.

### Inclusion criteria

**Fig. 1:**
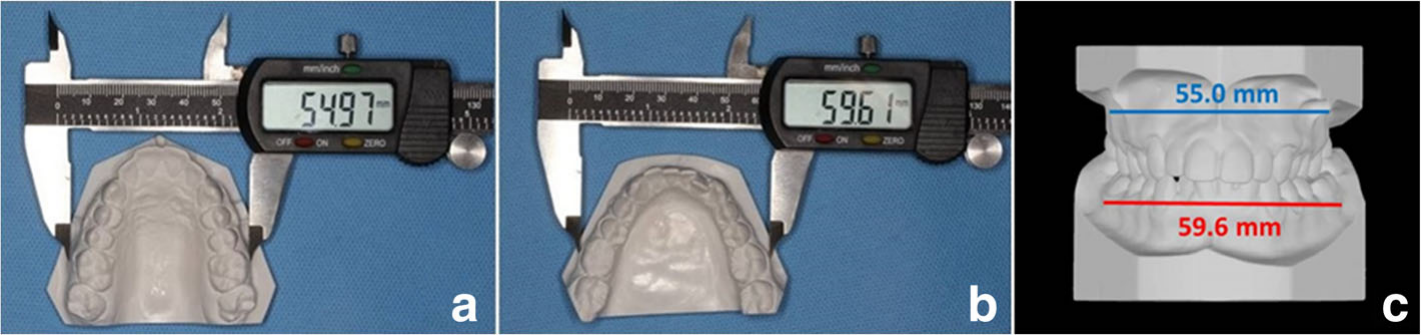
Measurement of maxillary (**a**) and mandibular width (**b**) with a digital caliper on the stone models. The frontal view of maxillary (blue line) and mandibular width (red line) is shown in (**c**). In this patient, the maxillary width is 55.0 mm, the mandibular width is 59.6 mm, and the maxillary transverse deficiency is 4.6 mm (59.6 − 55.0). The amount of maxillary skeletal expansion required for the patient is equal to the maxillary transverse deficiency (4.6 mm)

The inclusion criteria were the following: (1) transverse maxillary deficiency based on a modification of Andrews’ analysis of six elements [21], described below; (2) treatment with MSE as part of the overall treatment; (3) CBCT taken before and within 3 weeks of completion of active expansion; (4) absence of any craniofacial syndromes; and (5) lack of previous orthodontic treatment. Regarding point 1, the method adopted consists in analyzing the relationship between the maxillary and the mandibular width (Fig. 1). Maxillary width is defined as the distance between the right and left most concave point on maxillary vestibule at the level of the mesio-buccal cusp of first molars. Mandibular width is defined as the distance between the right and left mandibular WALA ridge at the level of the mesio-buccal groove of first molars. WALA ridge represents the most prominent portion of the buccal alveolar bone. In a normally developed maxilla, the maxillary width should be equal to the mandibular width. Maxillary transverse deficiency is calculated as the difference between mandibular and maxillary width, and represents the amount of maxillary skeletal expansion required for the patient, as shown in Fig. 1.

Patients were excluded from tooth-borne maxillary expansion and assigned to MSE treatment, based on the following criteria: patient maturity (appearance of secondary sexual characteristics such us facial hair, voice change, onset of menstruation cycle, cervical vertebral maturation stage higher than CS4) [22], dolicofacial vertical biotype (determined with SN-GoGn and FMA angles on lateral cephalometric analysis) and history of nasal airway obstruction. At UCLA Section of Orthodontics, dolicofacial patients are preferably treated with MSE rather than with a tooth-borne maxillary expander because, based on our experience, bone-borne appliances lead to lesser dentoalveolar tipping and lower posterior mandibular rotation.

### Expander design and activation rates

**Fig. 2:**
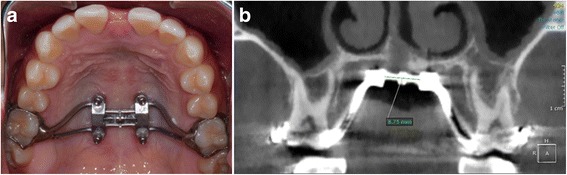
Maxillary Skeletal Expander (MSE). **a** Appliance positioned in the posterior part of the palate. **b** Measurement of the distance between the two halves of the expansion jackscrew on the post-expansion CBCT; the opening of the midpalatal suture can also be seen in the figure

MSE (Fig. 2a) elaboration and delivery was as proposed by Carlson [3]. The rate of expansion was two turns (.25 mm per turn) per day until inter-incisal diastema appeared and then one activation per day was applied. The expansion was completed when the maxillary width was equal to the mandibular width, as defined in Fig. 1. After the expansion, MSE remained blocked for at least 3 months to stabilize the expansion.

In order to calculate the average amount of activation of the expansion jackscrew received by the patients, the distance between the two halves of the expansion screw was measured on the post-expansion CBCT (Fig. 2b), and the pre-expansion distance, measured 10 times on a CBCT of the expansion device before activation, was then subtracted. The values were averaged to determine the mean and the standard deviation.

### 3D analysis

CBCT scans were taken before expansion and within 3 weeks of completion of active maxillary expansion on all patients. The duration of active maxillary expansion ranged between 12 and 36 days, while the average time period between the pre-expansion and post-expansion CBCT was 5 ± 2 months, as this included the time for insurance clearance, scheduling of appointments, appliance fabrication, and delivery. Secondary CBCT was taken before bonding of orthodontic brackets and within 3 weeks of completion of active expansion, in order to analyze suture openings before bone formation may occur. All CBCT scans were taken by a NewTom 5G scanner in an 18 × 16 field of view with a 14-bit gray scale and with a voxel size of 0.3 mm. Scan times were 18 s (3.6 s emission time), with 110 kV, and utilized an automatic exposure control that adjusted the milliampere based upon the patient’s anatomic density. Five hundred thirty-eight axial slices with 609 × 609 resolution and a slice thickness and increment of 0.3 mm and pixel spacing of 0.3 mm were obtained.

Utilizing the fusion module of OnDemand3D software from Cybermed Inc. company, the post-expansion CBCT was superimposed on pre-expansion CBCT using the anatomical structures of the whole anterior cranial base, as proposed by Cevidanes et al. for non-growing patients [19, 20]. The superimposition method utilizes the voxel gray scale and is fully automated by the software through the “Automatic Registration” tool, to avoid errors related to the operator. The accuracy of the method has been recently validated by Weissheimer et al. [23].

**Fig. 3:**
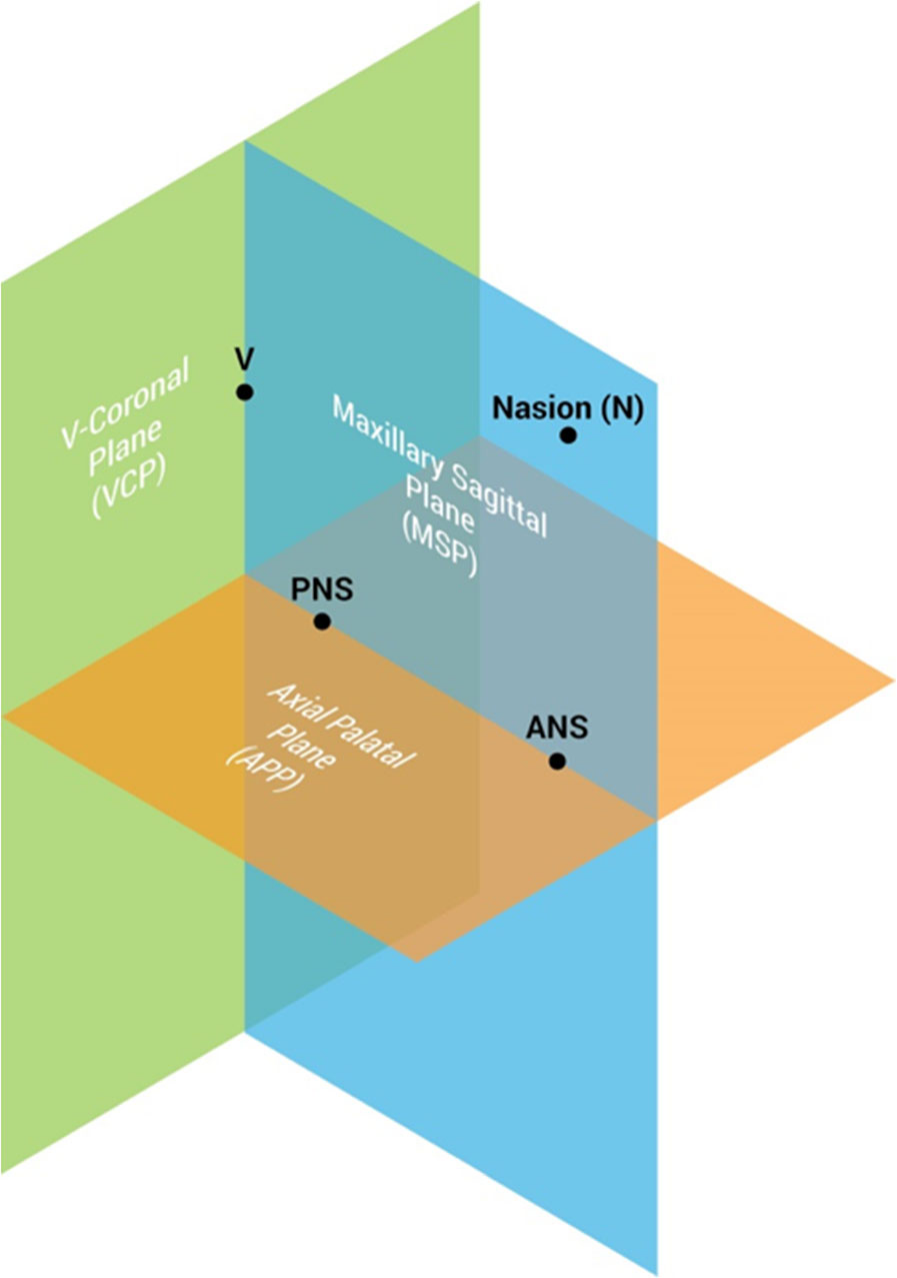
Schematic representation of the three main reference planes: maxillary sagittal plane (MSP), axial palatal plane (APP), and V-coronal plane (VCP). Reference planes are localized in the pre-expansion CBCT and become the reference lines to measure the displacement of skeletal structures in the post-expansion CBCT

**Fig. 4:**
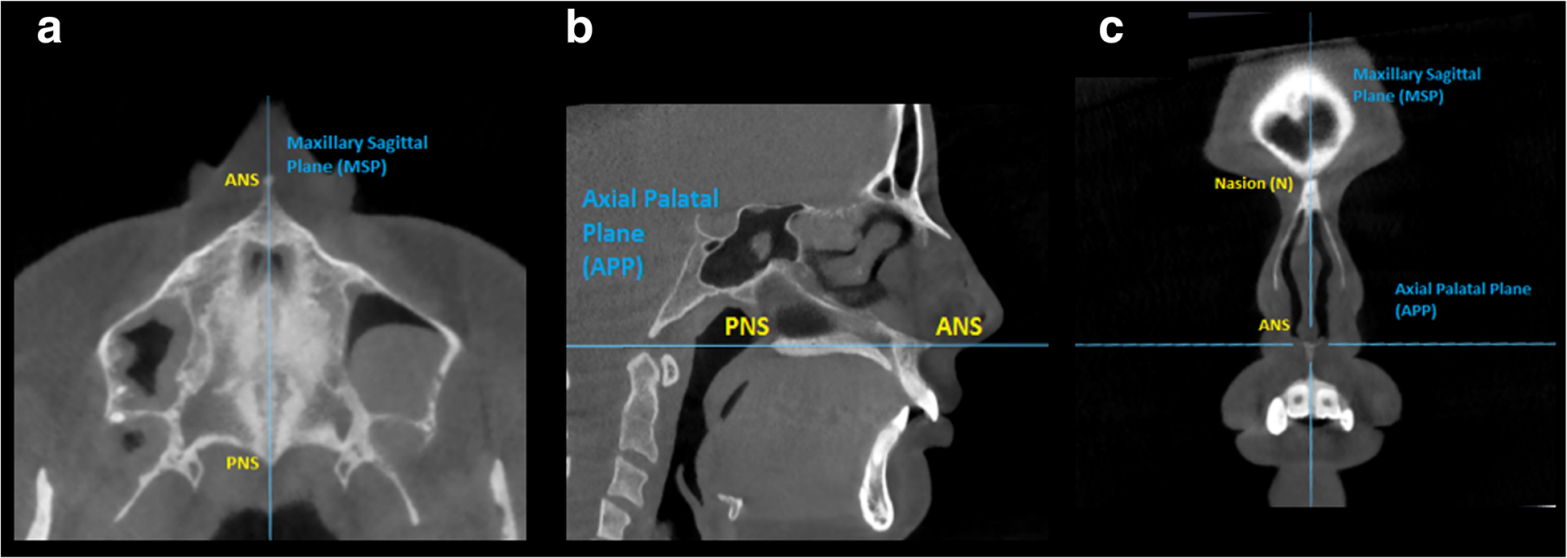
Sections used to localize the MSP and APP. **a** Axial section. **b** Sagittal section. **c** Coronal section

**Fig. 5:**
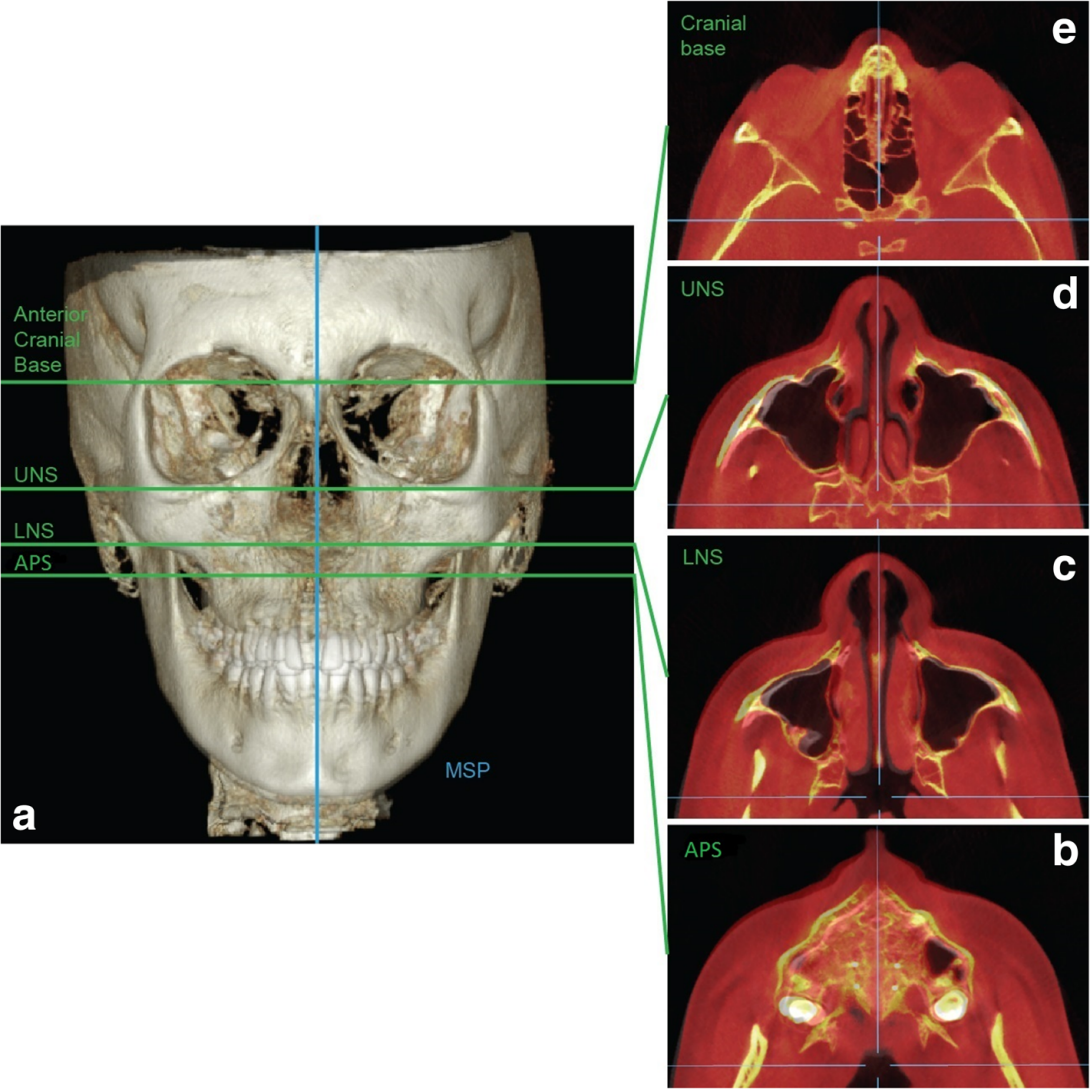
Axial palatal section (APS), lower nasal section (LNS), and upper nasal section (UNS) utilized to analyze the lateral and sagittal displacement of the maxilla and pterygoid plates and the modifications along the entire length of the pterygopalatine suture. **a** Superimposition of pre-expansion and post-expansion 3D model on anterior cranial base. **b** APS, superimposed image. **c** LNS, superimposed image. **d** UNS, superimposed image. **e** Anterior cranial base, superimposed image. It can be noticed how bones of the maxillo-facial complex are displaced in the APS, LNS, and UNS, while no displacement takes place on anterior cranial base. For this particular patient, skeletal changes are larger on the right side of the skull rather than on the left side, in all three axial sections (APS, LNS, UNS). Facial soft tissue modifications can also be detected on the APS, LNS, and UNS. Blue lines, MSP and VCP

Three novel reference planes have been identified in the present study in order to orient the skull: maxillary sagittal plane (MSP), axial palatal plane (APP), and V-coronal plane (VCP) (Fig. 3). Maxillary sagittal plane passes through the anterior nasal spine (ANS), posterior nasal spine (PNS), and nasion (N) on the pre-expansion CBCT; the axial palatal plane is perpendicular to the maxillary sagittal plane and passes through ANS and PNS (Fig. 4); the V-coronal plane is perpendicular to the other two planes and passes through the most posterior point of the vomer (V point). The three reference planes can be utilized to analyze the lateral, sagittal, and vertical displacement of the maxilla and surrounding structures induced by maxillary expansion. An axial section parallel to the axial palatal plane (APP) and passing through sella turcica was checked in every patient before taking the measurements in order to verify the accuracy of automated software superimposition on the anterior cranial base (Fig. 5e).

In order to describe the transverse and sagittal movement of the maxilla and pterygoid plates and the modifications in the pterygopalatine suture along its entire length, three axial sections have been analyzed: the axial palatal section (APS), lower nasal section (LNS), and upper nasal section (UNS) (Fig. 5). The axial palatal section (APS) passes through the axial palatal plane and is the section analyzed in this article. The lower nasal section (LNS) and upper nasal section (UNS) are parallel to the axial palatal section and will be the object of future publications.

### Measurements in the axial palatal section

**Fig. 6:**
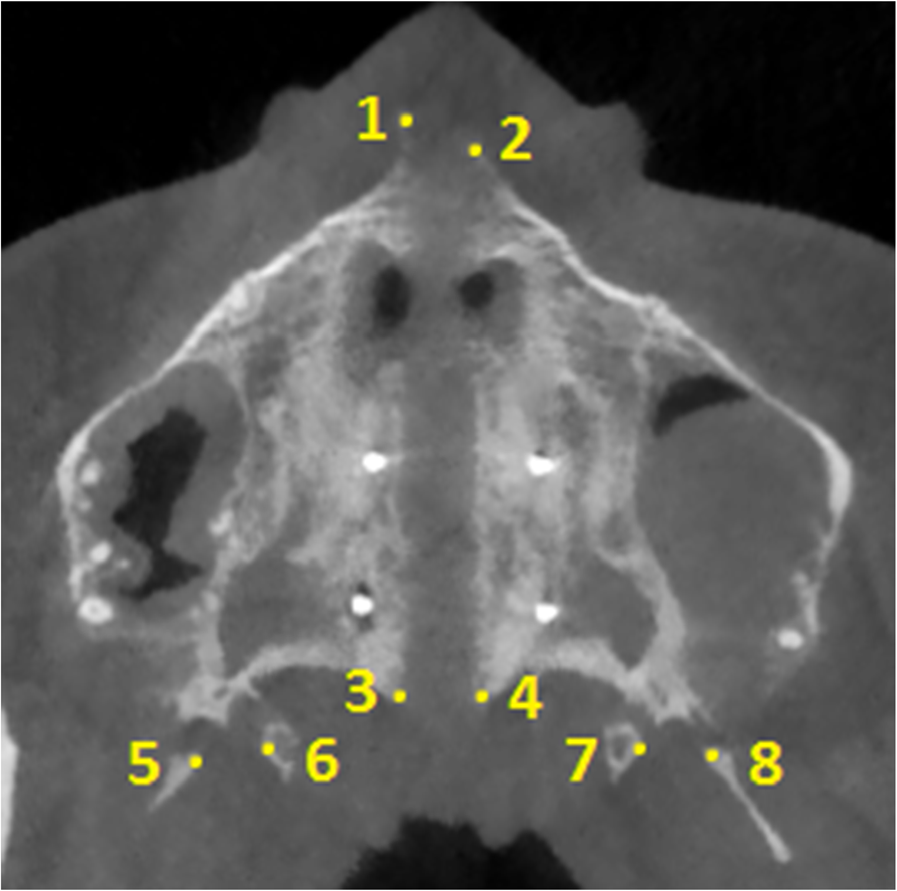
Landmarks identified in the axial palatal section in the post-expansion CBCT. 1 Right anterior nasal spine (Rt ANS), 2 left anterior nasal spine (Lt ANS), 3 right posterior nasal spine (Rt PNS), 4 left posterior nasal spine (Lt PNS), 5 most medial point of the lateral plate of the right pterygoid process (Rt Lat Pter), 6 most lateral point of the medial plate of the right pterygoid process (Rt Med Pter), 7 most lateral point of the medial plate of the left pterygoid process (Lt Med Pter), 8 most medial point of the lateral plate of the left pterygoid process (Lt Lat Pter)

**Fig. 7:**
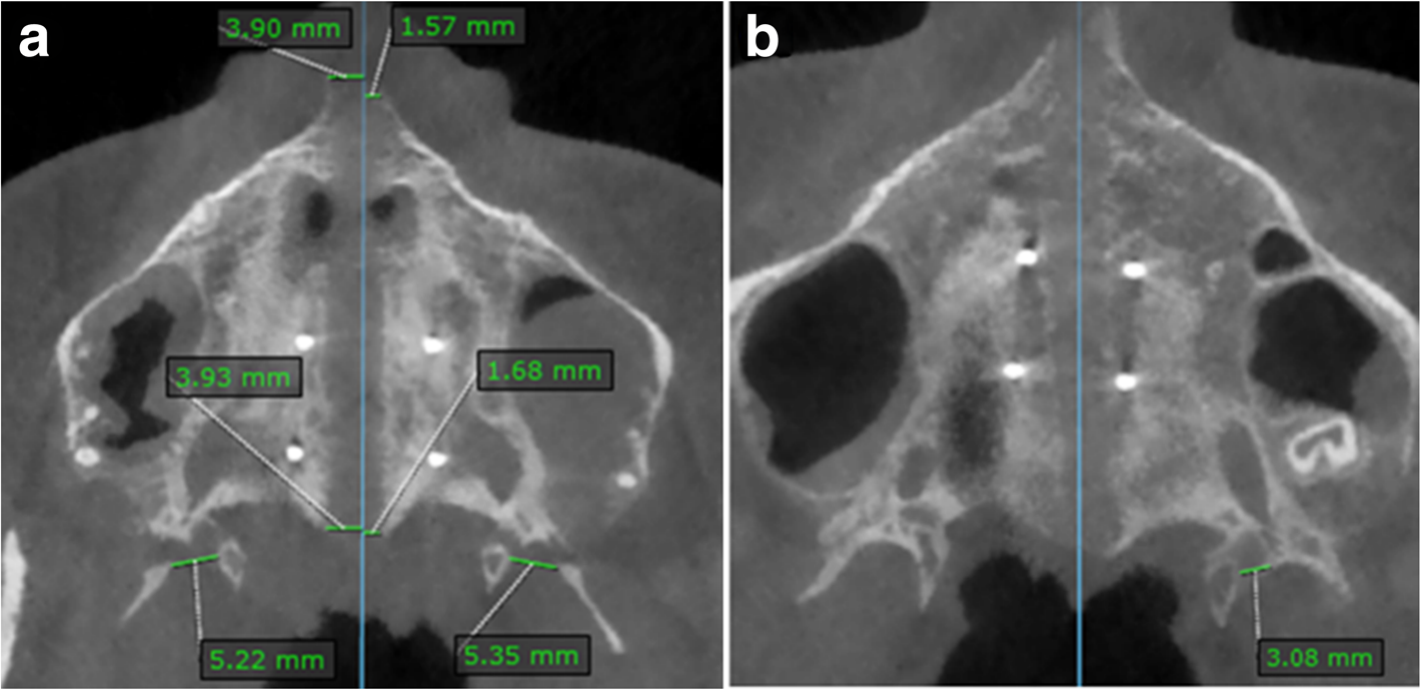
Measurements on axial palatal section on post-expansion CBCT. Blue line, maxillary sagittal plane. **a** Patient with a complete disengagement of the pyramidal process from the pterygoid plates. **b** Patient with a partial disengagement of the pyramidal process from the pterygoid plates in the left pterygopalatine suture; the opening is present between the pyramidal process and the medial pterygoid plate

In the present study, the APS has been used to study the split of the midpalatal and pterygopalatine sutures. The utilized landmarks are shown in Fig. 6, and the evaluated parameters were the following: distance of right and left half of ANS and PNS from maxillary sagittal plane, sum of lateral displacement of Rt ANS and Lt ANS, sum of lateral displacement of Rt PNS and Lt PNS, and width of opening in right and left pterygoid processes (Fig. 7). In addition, the frequency of openings in the pterygoid processes was calculated.

The split of the midpalatal suture generates two halves of the ANS and PNS. The maxillary sagittal plane passes through the ANS and PNS in the pre-expansion CBCT, and the distance between the two halves from the maxillary sagittal plane in the post-expansion CBCT represents the lateral movement of each half (Fig. 7a).

The extent of transverse asymmetry of the split of the midpalatal suture was calculated as follows: for each patient, the lesser movement of one half of ANS was subtracted from the larger movement of the contralateral half. The values were then averaged. This parameter shows on average how much more one half moves with respect to the contralateral half. The movement of the ANS and not the PNS was chosen as a parameter to evaluate the asymmetry because changes at ANS reflect modifications in the anterior part of the maxilla more closely and therefore can have a larger impact on the soft tissues of the face (Fig. 5b–d).

**Fig. 8:**
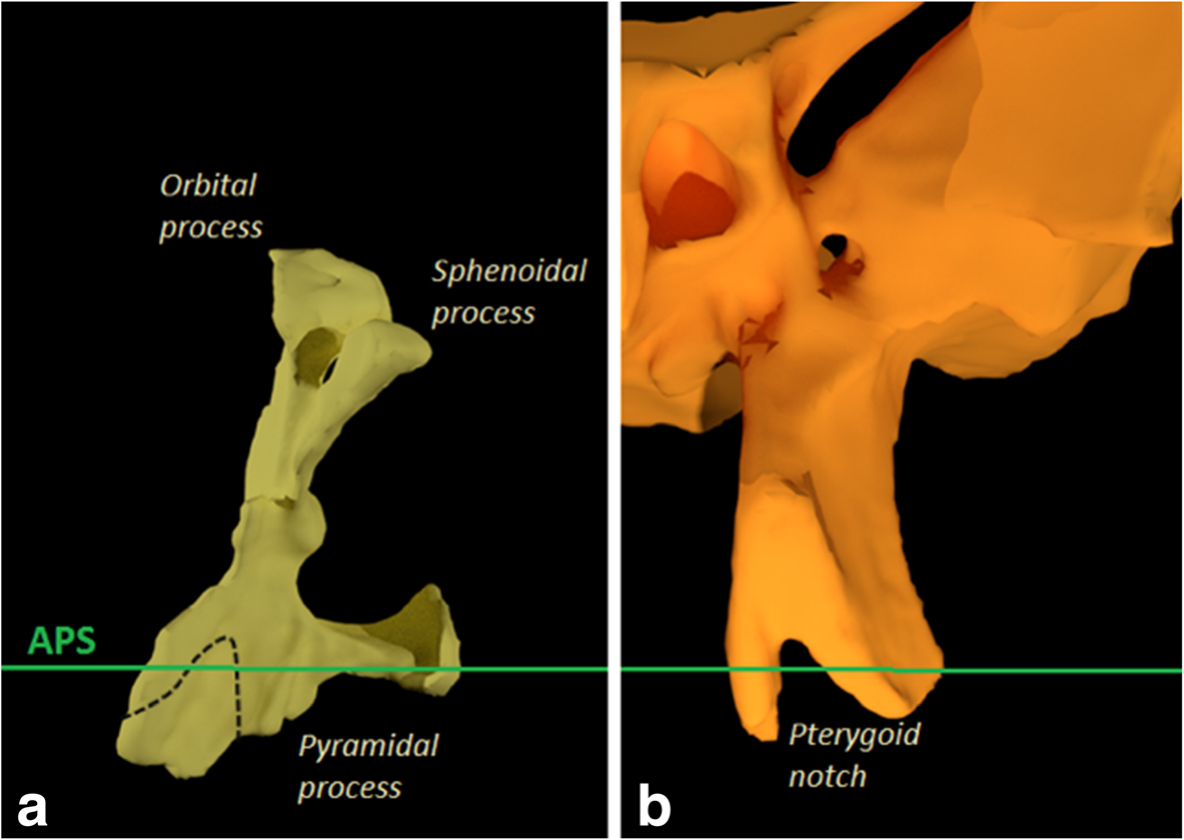
Relation between the APS with palatine bone and pterygoid plates. **a** Posterior view of the left palatine bone. **b** Anterior view of the left pterygoid process of the sphenoid bone

Furthermore, the axial palatal section cuts the pterygopalatine suture in an area where the pyramidal process of the palatine bone articulates with the pterygoid notch located between the lateral and the medial plate of the pterygoid process (Fig. 8). Changes in this area due to the maxillary expansion are described as “openings” between the lateral and medial pterygoid plates.

The average width and frequency of the openings (i.e., percentage of sutures presenting the openings) were calculated. The width of the opening between the lateral and medial pterygoid plates was measured as the distance from the most medial point of the lateral pterygoid plate to the most lateral point of the medial pterygoid plate (Figs. 6 and 7). In the case of a partial disengagement of the pyramidal process from the pterygoid notch, the width of the opening was measured from the pyramidal process to the pterygoid plate adjacent to the opening (Fig. 7b). The whole extension of articulation between the pyramidal process of the palatine bone and the pterygoid notch of the pterygoid process was analyzed with consecutive axial sections parallel to the axial palatal plane for each patient, and the section with the widest opening was chosen as the measurement. In the pre-expansion CBCT, the frequency and width of the openings were given a value equal to zero.

The landmarks shown in Fig. 6 could be detected only in the post-expansion CBCT since in the pre-expansion CBCT, the two halves of ANS and PNS are in contact. In addition, there are no gaps between the lateral and medial plates of the pterygoid processes since the pterygoid notch is occupied by the pyramidal process of the palatine bone (Fig. 8).

### Statistical analysis

Sample size analysis was performed for 80% power with an alpha value of 0.05.

The assessments of parameters were compared from pre- to post-treatment non-parametrically using the Wilcoxon sign rank test. A non-parametric test was chosen because the pre-expansion values of all considered parameters were equal to zero (non-normal distribution). The Wilcoxon sign rank test was also utilized to compare the magnitude of suture openings between males and females.

The Fisher’s exact test was used to compare the frequency of openings in the lower part of the pterygopalatine suture from pre-expansion to post-expansion, and between males and females.

A correlation analysis between age and parameters of suture opening was performed.

Reliability of skull orientation was evaluated by re-orienting 30% of randomly selected CBCT scans (*n* = 5 patients) and calculating the intra-class correlation coefficient, using the posterior clinoid process and basion as landmarks for comparison, similarly to the method adopted by Woller et al. [24].

Reliability of measured parameters was obtained on 10 different patients by two raters. Each parameter was measured twice by each rater. Reliability was evaluated by computing the intra-class correlation coefficient (ICC) *ρ*.

## Results

The sample sizes needed for 80% power are given in Table 1. The sample size of 15 provided at least 80% power for all considered parameters, for confirming treatment change using the usual 0.05 two-sided significance level.

**Table 1:** Sample size analysis for 80% power, with 0.05 alpha value

Parameter	Number	Treatment change		*n* for 80% power
		Mean	sd	
Rt ANS to maxillary sagittal plane	15	2.59	1.70	6
Lt ANS to maxillary sagittal plane	15	2.16	1.27	5
Rt PNS to maxillary sagittal plane	15	2.34	1.32	5
Lt PNS to maxillary sagittal plane	15	1.99	0.90	4
Width of opening in Rt pterygoid process	15	1.35	1.79	15
Width of opening in Lt pterygoid process	15	2.17	2.45	13

The average amount of activation of MSE expansion jackscrew was 6.8 ± 1.9 mm (range, 4.1–10.5 mm). The duration of maxillary expansion ranged from 12 to 36 days.

Regarding the midpalatal suture, the amount of split at PNS (4.3 mm) was 90% of that at ANS (4.8 mm), as shown in Table 2.

**Table 2:** Results for lateral displacement of ANS and PNS and width of opening in pterygoid processes

		Before expansion		After expansion		Treatment change		*P* value	
		Mean	sd	Mean	sd	Mean	sd		
1	Rt ANS to maxillary sagittal plane	0.00	0.00	2.59	1.70	2.59	1.70	< .0001	**
2	Lt ANS to maxillary sagittal plane	0.00	0.00	2.16	1.27	2.16	1.27	< .0001	**
3	Rt PNS to maxillary sagittal plane	0.00	0.00	2.34	1.32	2.34	1.32	< .0001	**
4	Lt PNS to maxillary sagittal plane	0.00	0.00	1.99	0.90	1.99	0.90	< .0001	**
5	Lateral displacement of Rt ANS + Lt ANS	0.00	0.00	4.75	2.59	4.75	2.59	< .0001	**
6	Lateral displacement of Rt PNS + Lt PNS	0.00	0.00	4.33	1.74	4.33	1.74	< .0001	**
7	Width of opening in Rt pterygoid process	0.00	0.00	1.35	1.79	1.35	1.79	0.011	*
8	Width of opening in Lt pterygoid process	0.00	0.00	2.17	2.45	2.17	2.45	0.004	**

All measures are expressed in mm

*Rt ANS* right half of anterior nasal spine, *Lt ANS* left half of anterior nasal spine, *Rt PNS* right half of posterior nasal spine, *Lt PNS* left half of posterior nasal spine, *Rt* right, *Lt* left

*P < .05; **P < .01

The split of the midpalatal suture was asymmetrical: on average, one half of the ANS moved more than the contralateral half by 1.1 (± 1.0) mm.

With regard to the pterygopalatine suture split, 16 sutures out of 30 (53%) presented openings between the medial and lateral pterygoid plates (*P* < .01).

No significant differences were found in the magnitude and frequency of suture opening between males and females, as shown in Table 3.

**Table 3:** Analysis of differences in suture opening between males and females

	Males		Females		*P* value
	Mean	sd	Mean	sd	
MSE activation	7.48	1.26	6.36	2.20	0.124
Lateral displacement of Rt ANS + Lt ANS	5.26	3.14	4.42	2.31	0.556
Lateral displacement of Rt PNS + Lt PNS	4.69	2.42	4.09	1.21	0.557
Frequency of split in pterygopalatine suture	7/12 (58.3%)		9/18 (50.0%)		0.722

*Rt ANS* right half of anterior nasal spine, *Lt ANS* left half of anterior nasal spine, *Rt PNS* right half of posterior nasal spine, *Lt PNS* left half of posterior nasal spine

The correlation analysis between age and parameters of suture opening is given in Table 4. Correlation was negligible for all considered parameters.

**Table 4:** Analysis of correlation between age and parameters of suture opening

Parameter	Spearman correlation coefficient	*P* value	*R* ^2^ (%)
Lateral displacement of Rt ANS + Lt ANS	− 0.204	0.466	4.2
Lateral displacement of Rt PNS + Lt PNS	− 0.148	0.599	2.2
Frequency of split in pterygopalatine suture	0.055	0.845	0.3

Skull orientation was highly reliable, as the obtained Cronbach’s alpha value was 0.987.

For the considered parameters, the intra-class correlation coefficient (ICC) *ρ* was at least 0.973 or more, showing that measurements were very reliable (Table 5).

**Table 5:** Intra-class correlation coefficients (ICC) of the parameters. *Rt ANS* right half of anterior nasal spine, *Lt ANS* left half of anterior nasal spine, *Rt PNS* right half of posterior nasal spine, *Lt PNS* left half of posterior nasal spine, *Rt* right, *Lt* left

Parameter	ICC
Distance of Rt ANS from maxillary sagittal plane	0.980
Distance of Lt ANS from maxillary sagittal plane	0.984
Distance of Rt PNS from maxillary sagittal plane	0.981
Distance of Lt PNS from maxillary sagittal plane	0.976
Width of opening in Rt pterygoid process	0.973
Width of opening in Lt pterygoid process	0.984
Distance between two halves of expansion screw	0.979

## Discussion

**Fig. 9:**
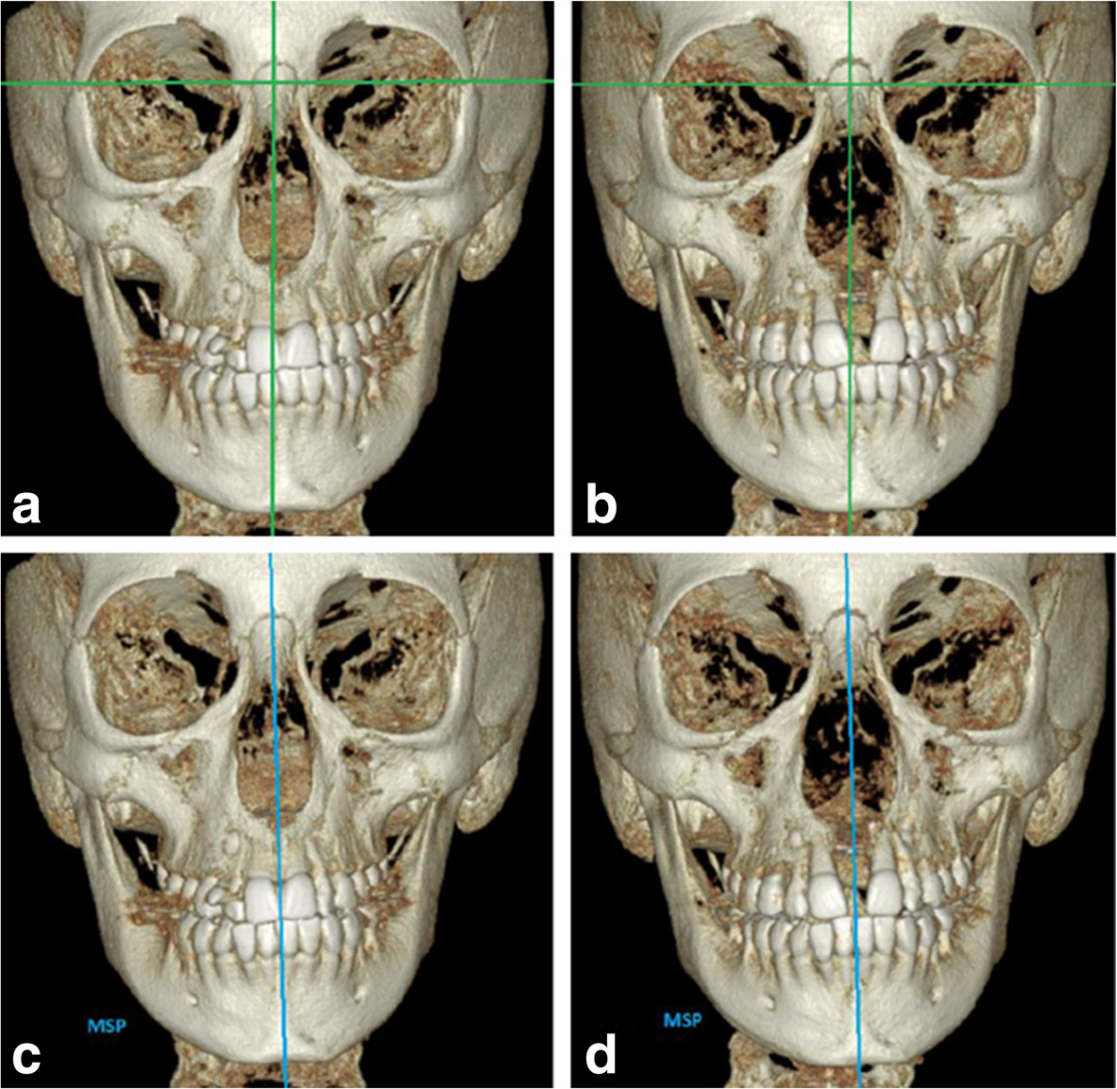
Reference lines used in conventional 2D postero-anterior cephalometric analysis: **a** pre-expansion and **b** post-expansion; ANS is located on the left side of the vertical reference line. Maxillary sagittal plane (MSP) as novel reference plane: **c** pre-expansion and **d** post-expansion; ANS is located in the MSP

For the evaluation of facial structures in the frontal plane, 2D postero-anterior cephalometric analyses commonly utilize a reference line perpendicular to a line connecting the right and left frontozygomatic sutures and passing through Crista Galli of the ethmoid bone (Fig. 9a, b), and reference planes based on the cranial base have been described also in 3D cephalometric analyses [25]. However, reference lines or planes based on the cranial base do not necessarily cross the midpalatal suture so that ANS or PNS fall either on the one side or on the other side of the vertical reference line/plane. In the present study, the maxillary sagittal plane (MSP) has been developed in order to establish a procedure that allows to analyze the extent of transverse asymmetry of the split of the midpalatal suture and the movement of each maxillary bone (Fig. 9c, d).

**Fig. 10:**
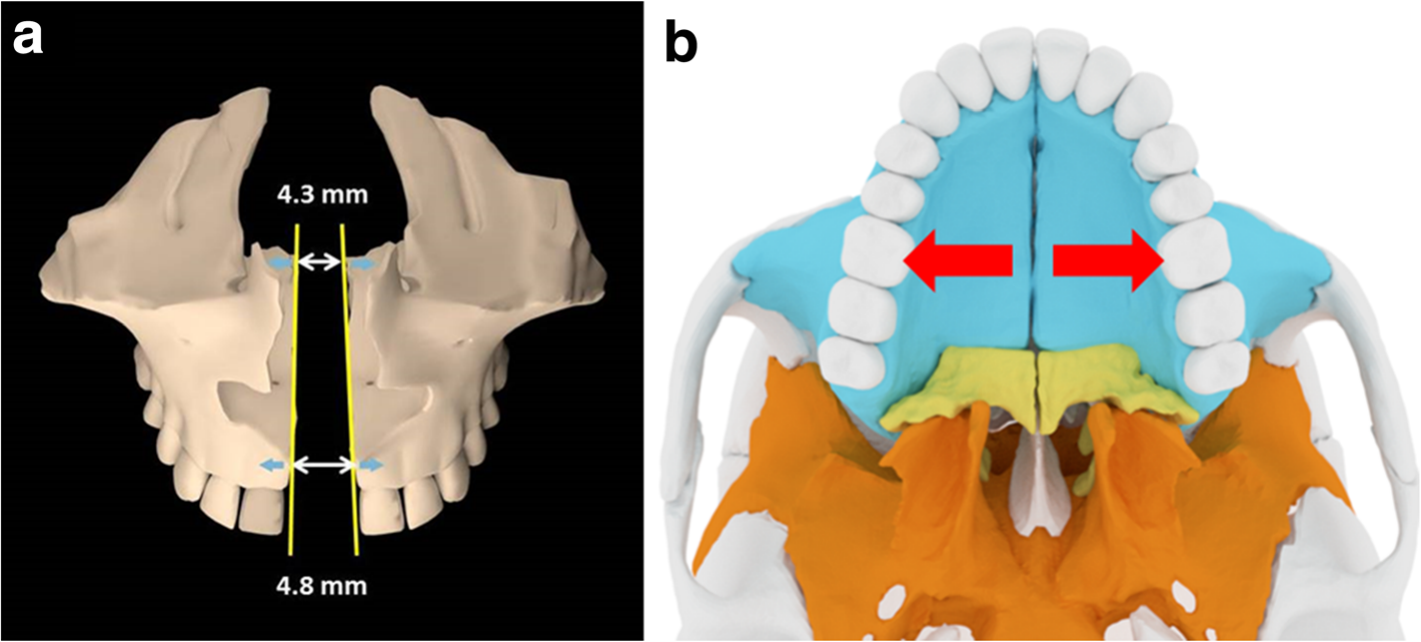
Sagittal parallelism of midpalatal suture opening obtained with MSE. **a** Borders of the midpalatal suture (yellow lines) moving almost perfectly parallel to each other; amount of split at PNS (4.3 mm) was 90% of that at ANS (4.8 mm). **b** Position of MSE in the posterior part of the palate, providing an expansion force (red arrows) in line with the zygomatic buttress bones, which represent a major resistance to the movement of maxillary halves

Regarding the midpalatal suture, this was successfully split by MSE in all patients in the present study. In the literature, it is described that tooth-borne maxillary expanders produce a V-shaped opening of the midpalatal suture, with the greatest opening anteriorly and progressively less separation towards its posterior part [12, 18, 26]. Lione et al. [18] utilized a tooth-borne maxillary expander activated by 7 mm on all patients and found that the opening of the midpalatal suture was 3.01 and 1.15 mm at ANS and PNS, respectively. The split at PNS was about 40% of that at ANS, showing the V-shaped expansion pattern. Conversely, in the patients treated with MSE in the present study, the borders of the midpalatal suture moved almost perfectly parallel to each other since the amount of split at PNS (4.3 mm) was 90% of that at ANS (4.8 mm) (Fig. 10a). This is probably due to the different biomechanics in MSE compared to tooth-borne maxillary expanders [3–5]. The use of four mini-implants in the MSE, with a considerable antero-posterior distance between them and positioned in the posterior part of the palate, medial to the zygomatic buttress bones, allows the separation force to be distributed along the entire suture length (Fig. 10b). This promotes the more parallel split of the midpalatal suture in an antero-posterior direction [27].

Furthermore, the magnitude of the separation of the midpalatal suture with MSE was larger, compared to the findings of Lione et al. [18] for a modified Hyrax-type expander. In the present study, the expansion at the level of the midpalatal suture was 71 and 63% at ANS (4.8 mm) and PNS (4.3 mm), respectively, compared to the amount of activation of the jackscrew (6.8 mm). This ratio is higher than what has been reported by Lione et al. (43% for ANS and 16% for PNS) [18], probably due to the different anchorage modality of the expansion device (bone-borne versus tooth-borne). In fact, dentoalveolar tipping is an important modification induced by tooth-borne expanders since the expansion force is transmitted to the maxillary bones via the dentition [18, 28], while it is negligible with MSE, even in late adolescents, as shown in a recent case report [3].

**Fig. 11:**
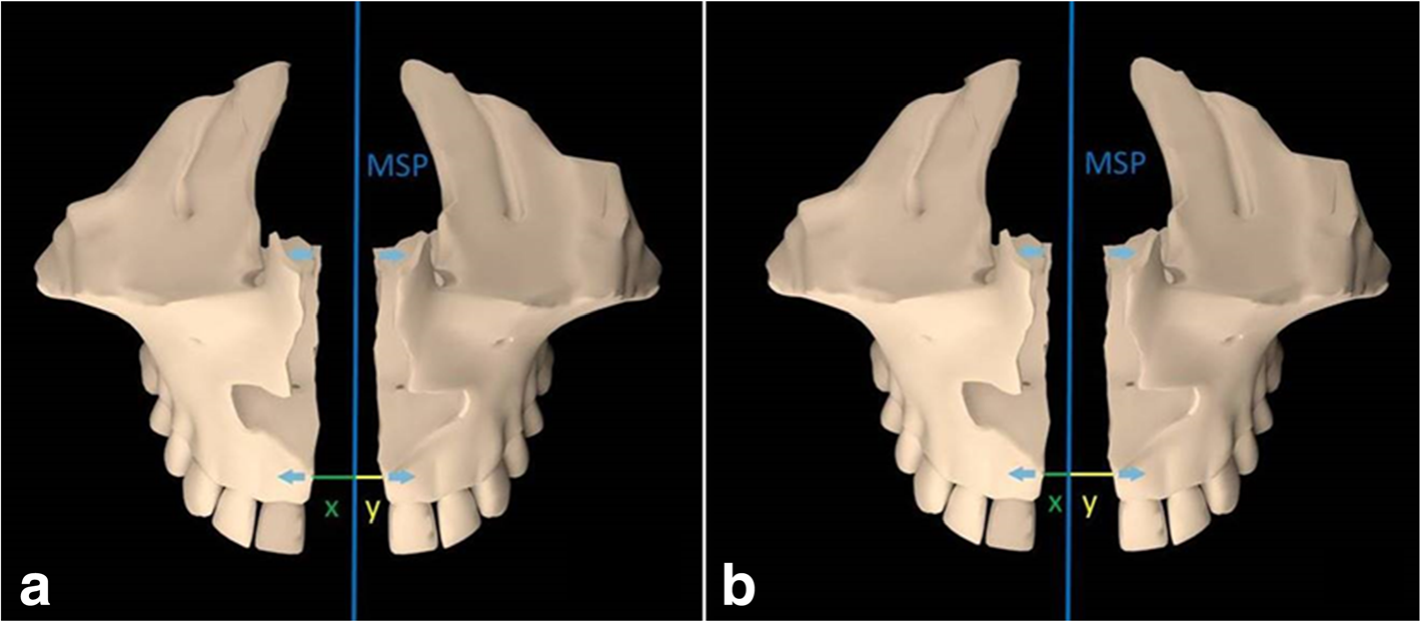
Schematic representation of transverse asymmetry of split of the midpalatal suture. **a** Example where the lateral movement of right maxilla (x) is larger than that of left maxilla (y). **b** Example where the lateral movement of left maxilla (y) is larger than that of right maxilla (x). Blue line, MSP

Analyzing the transverse asymmetry of midpalatal split (Fig. 11), on average one half of the ANS moved more than the contralateral one by 1.1 mm. The reason why the midpalatal suture splits with an asymmetrical pattern is unknown. One reason could be related to external forces, such as the presence of a unilateral crossbite that hampers the movement of one maxilla. Another hypothesis could be associated with circummaxillary sutures. These sutures may not become loose in the same proportion in both sides of the skull during RPE. Thus, one maxillary bone could present more lateral displacement and therefore explain asymmetrical expansion. In addition to circummaxillary sutures, also discrepancies in zygomatic buttress bone density and morphology can play an important role in this phenomenon. The movement of the anterior part of maxillary bones can affect soft tissues in the midface during maxillary expansion [29], and this might lead to esthetic modifications in this region that could become asymmetrical. The analysis of the transverse asymmetry of midpalatal suture split was performed for the first time in our study. Further investigations with tooth-borne maxillary expanders and different MARPE designs are recommended.

With regard to the pterygopalatine suture, Ghoneima et al. in a clinical investigation conclude that the cited suture cannot be split when tooth-borne expanders are used for rapid palatal expansion [11]. However, MSE has shown that the pterygopalatine suture was split, as the pyramidal process of the palatine bone was pulled away from the medial and lateral plates of the pterygoid process, leaving detectable openings on the CBCT in 16 out of 30 sutures (53%) (Fig. 7). Seven patients showed openings in both the right and left pterygopalatine sutures, one patient in the right suture only, and one patient in the left one only.

The average width of openings between pterygoid plates was 1.4 and 2.2 mm for the right and left suture, respectively. The width depends on the size of the pyramidal process of the palatine bone that presents large variability in shape and size among the population [30]. Also, if partial disengagement of the pyramidal process from the pterygoid process takes place, such in the case of a minor movement of the maxilla, the opening is not complete and hence presents a smaller width (Fig. 7b). In the case of a complete disengagement, the width becomes larger (Fig. 7a). The complete disengagement of the pyramidal process from the pterygoid plates was the most common finding, as it was present in 13 sutures, while the partial disengagement was detected in 3 sutures only. From the data above, it can be inferred that when the pterygopalatine suture became disarticulated, it was most commonly split bilaterally on the right and left side of the skull and with a complete disengagement of the pyramidal process from the pterygoid plates. The reason why the pterygopalatine suture was disarticulated in some patients and not in other can be related to anatomical factors such as the size and shape of the pyramidal process that are largely variable [30] and hence can influence the resistance to the suture split. Other reasons can be the individual bone density and thickness, the degree of suture interdigitation, or complex interactions among various circummaxillary sutures. In the present study, the patient gender and age had no significant influence on the frequency of pterygopalatine suture split.

The finding of an almost perfectly parallel split of the midpalatal suture with MSE indicates a large movement of the posterior part of the maxilla that leads to the disarticulation of the pyramidal process of the palatine bone from the pterygoid process of the sphenoid. Conversely, the typical V-shaped split of the midpalatal suture in Hyrax patients is associated with a lack of disarticulation of the pterygopalatine suture, as reported in a clinical study [11]. This posterior skeletal expansion with MSE can offer certain advantages such as improving posterior occlusion in patients with maxillary deficiency, providing patent nasal airway with relieve of posterior constriction. These findings can have important implications also in the treatment of class III patients when MSE is followed by facemask therapy; MSE loosens the pterygopalatine suture and can reduce its resistance to maxillary protraction. In fact, rapid palatal expansion is a procedure commonly utilized to enhance maxillary advancement.

The correlation between age and parameters of suture opening was negligible, as the *R* ^2^ coefficient for the considered parameters ranged from 0.3 to 4.2%. This can be due to the fact that the analyzed patients were at post-pubertal age, when most significant changes in suture interdigitation have already occurred [10].

One limitation of the present study is the small number of patients. The statistical analysis showed that a minimum sample size of 15 is needed to show the effects on sutures with at least 80% power for the considered parameters. However, further studies with a larger sample would be beneficial.

## Conclusions

- ▪ The novel reference planes and novel methodology allowed for quantification of the opening of the midpalatal and pterygopalatine sutures produced by maxillary expansion; this method also made it possible to evaluate the extent of transverse asymmetry of split of the midpalatal suture.
- ▪ MSE efficiently split the midpalatal suture in late adolescents, and separation at posterior nasal spine (4.3 mm) was about 90% of that at anterior nasal spine (4.8 mm), leading to an almost perfectly parallel split of the suture in the sagittal direction.
- ▪ The split of the midpalatal suture was asymmetrical in the transverse direction; on average one half of ANS moved more than the contralateral one by 1.1 mm.
- ▪ Remarkably, this study shows that the pterygopalatine suture can be split by an orthopedic appliance without the need of surgery in late adolescents; MSE was able to split the pterygopalatine suture in its lower part in 53% of the sutures, as the pyramidal process of the palatine bone was pulled out of the pterygoid notch of the pterygoid process.
- ▪ Patient gender and age had a negligible influence on magnitude and frequency of sutures opening for the age group considered in the study.

## Abbreviations

ANS: Anterior nasal spine
APP: Axial palatal plane
CBCT: Cone beam computed tomography
ICC: Intra-class correlation coefficient
IRB: Institutional Review Board
MARPE: Micro-implant-assisted rapid palatal expansion
MSE: Maxillary Skeletal Expander
MSP: Maxillary sagittal plane
N: Nasion
PNS: Posterior nasal spine
RME: Rapid maxillary expansion
RPE: Rapid palatal expansion
SARPE: Surgically assisted rapid palatal expansion
V point: Most posterior point of the vomer
VCP: V-coronal plane
